# Application of nanomedicine in radiotherapy sensitization

**DOI:** 10.3389/fonc.2023.1088878

**Published:** 2023-02-15

**Authors:** Xiaoyu Song, Zhenkun Sun, Li Li, Lu Zhou, Shuanghu Yuan

**Affiliations:** ^1^ School of Clinical Medicine, Weifang Medical University, Weifang, China; ^2^ Department of Radiation Oncology, Shandong Cancer Hospital and Institute, Shandong First Medical University and Shandong Academy of Medical Sciences, Shandong Cancer Hospital Affiliated to Shandong First Medical University, Jinan, China; ^3^ Shunde Hospital, Guangzhou University of Traditional Chinese Medicine, Foshan, Guangdong, China; ^4^ Department of Radiation Oncology, Shandong Cancer Hospital Affiliated to Shandong University, Jinan, China; ^5^ Department of Radiation Oncology, The Affiliated Cancer Hospital of Zhengzhou University, Zhengzhou, China

**Keywords:** radiotherapy, therapeutics, radiosensitizers, nanomedicine, mechanism, nano-radiosensitizers

## Abstract

Radiation therapy is an important component of cancer treatment. As research in radiotherapy techniques advances, new methods to enhance tumor response to radiation need to be on the agenda to enable enhanced radiation therapy at low radiation doses. With the rapid development of nanotechnology and nanomedicine, the use of nanomaterials as radiosensitizers to enhance radiation response and overcome radiation resistance has attracted great interest. The rapid development and application of emerging nanomaterials in the biomedical field offers good opportunities to improve the efficacy of radiotherapy, which helps to promote the development of radiation therapy and will be applied in clinical practice in the near future. In this paper, we discuss the main types of nano-radiosensitizers and explore their sensitization mechanisms at the tissue level, cellular level and even molecular biology and genetic level, and analyze the current status of promising nano-radiosensitizers and provide an outlook on their future development and applications.

## 1 Introduction

Cancer is the leading cause of death worldwide. The number of effective methods for diagnosing and treating cancer is increasing day by day. The main traditional tumor-based treatments currently available include surgery, chemotherapy and radiotherapy (RT). In addition, modern treatments have emerged in recent years, such as immunotherapy, gene therapy, photodynamic therapy (PDT), photothermal therapy (PTT), chemodynamic therapy (CDT), etc. Radiotherapy is one of the most widely employed methods clinically, roughly half of all cancer patients undergo some form of RT during the course of their treatment, either alone or in combination with surgery or chemotherapy. Radiation therapy uses ionizing radiation (IR) to induce DNA damage, such as DNA single-strand breaks (SSB) or double-strand breaks (DSB) and DNA-DNA or DNA-protein cross-links (DPCs), which are important mechanisms for tumor killing (1).

Although radiotherapy is highly effective, it still needs to be used with caution. The efficacy of radiotherapy is closely related to the dose of radiation. Patients can be treated with high doses of radiation to enhance the effects of radiation therapy, but serious side effects are inevitable. While killing cancer cells, it will cause severe damage to the normal tissues penetrated by radiation rays (2). Reducing the radiation dose may improve patient compliance, but may compromise the efficacy of the treatment and fail to eliminate the tumor completely. In addition, another vexing limitation of low-dose radiation therapy is the possibility of radiation resistance, leading to the failure of RT (3).

In recent years, research on the causes and mechanisms of action of tumor radiotherapy resistance has been increasing, and abnormalities in signaling pathways such as PI3K/Akt, Wnt/β-catenin, ATM, NF-κB and MAPK have been associated with radiotherapy resistance. In-depth studies of these signaling pathways will provide new strategies to improve the efficacy of RT (4). Hence, new approaches to improving the response of tumors to radiation need to be put on the agenda in order to achieve therapeutic results at low radiation doses.

Radiation sensitizers play an important role in radiotherapy and when combined with radiation, the tumor inactivation effect obtained is greater than the expected additive effect of each modality. New targets and mechanisms of radiosensitization are being discovered, opening up new avenues for the development and application of radiosensitization agents for tumors (5). The latest view is that radiosensitizers can be classified into three major categories according to their different structures, namely small molecules, macromolecules and nanomaterials (4).

**Figure 1:**
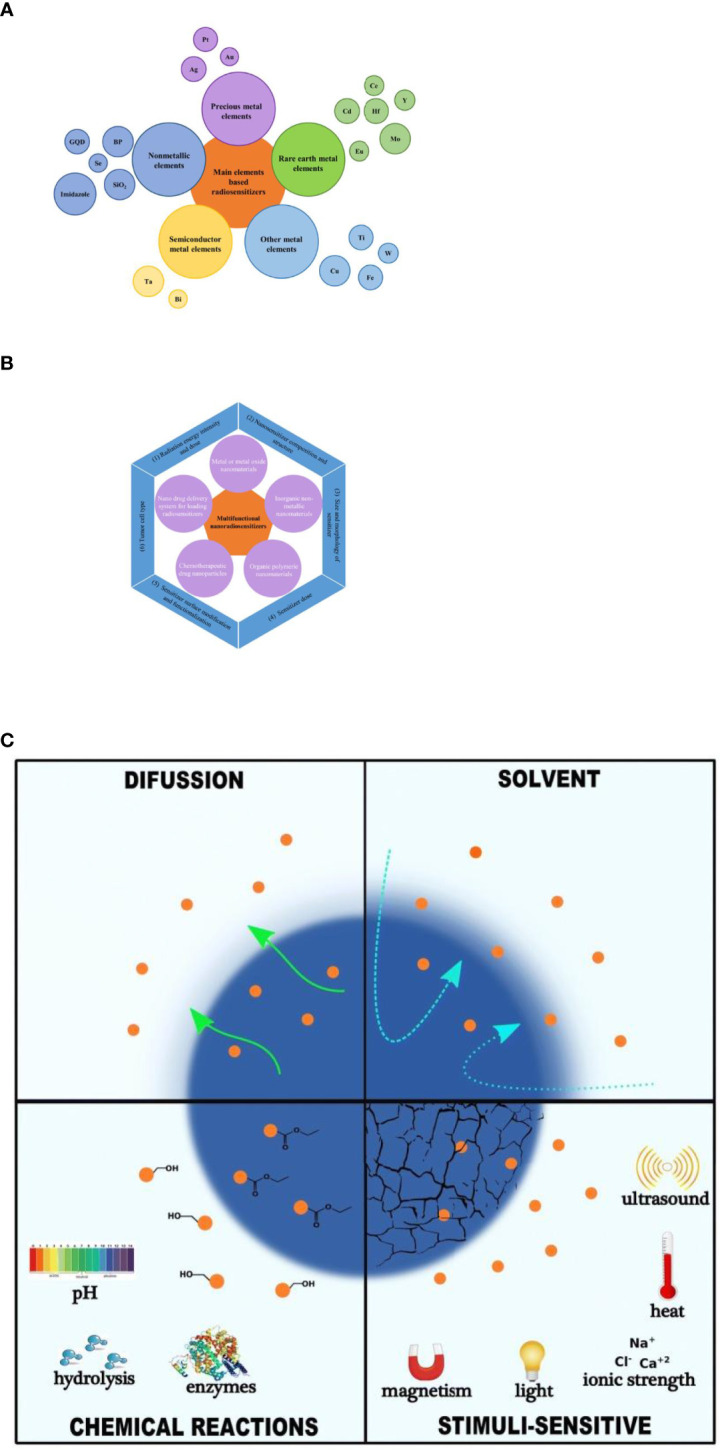
Classification related to nanoradiotherapy sensitization. **(A)** Classification of main element-based nanomaterials as radiosensitizers. **(B)** Main types and influencing factors of nano-radiosensitizers. **(C)** Mechanisms of controlled drug release using different types of nanocarriers. This research was originally published in (9). Copyright(2018) Recent Patents on Drug Delivery & Formulation.

The use of nanotechnology and nanomedicine for cancer radiotherapy has grown more and more (6, 7). A large number of nanomaterials have been developed as radiosensitizers to enhance local therapeutic effects and reduce adverse effects (8). The introduction of nanotechnology provides a driving force for the development of radiosensitizers and expands the field of vision. The types of nanomaterials are not limited to precious metals (silver (Ag), gold (Au), and platinum (Pt)); some nanomaterials that are based on rare earth metals (gadolinium (Gd), hafnium (Hf), etc.), semiconductor metals (bismuth (Bi)), and other metals (titanium (Ti), etc.) and non-metal nano-radiosensitizers are also widely used (**Figure 1A**).

In this paper, we summarize the main types of nano-radiosensitizers and examples that have been studied in the clinical setting, as well as the main routes of action and influencing factors, taking into account the advantages and limitations of the nano-radiosensitizers. Finally, the future development and application of nano-radiosensitizers is foreseen.

## 2 Categories of nanomaterials applied in radiotherapy

With the rapid development of nanotechnology and nanomedicine, nanomaterials have attracted strong interest in enhancing radiation responses and overcoming radio-resistance due to a variety of physicochemical properties such as good biocompatibility, intrinsic radiosensitive activities, highly loading abilities of multiple types of drugs and the enhanced permeability and retention (EPR) effects in tumor tissue. Nanomaterials can be generally divided into the following types (**Figure 1B**).

### 2.1 Metal or metal oxide nanomaterials

Metal nanoparticles are novel radiosensitizers due to high absorption coefficient, biocompatibility, synthetic versatility and unique chemical, electronic and optical properties. The relationship between the X-ray absorption phenomenon (E) and the atomic number (Z) is as follows: µ = *ρZ*4/(*AE*3), where ρ is the density, A is the atomic mass of the element and μ is the X-ray absorption coefficient (μ) (10). Therefore, the X-ray absorption coefficient (μ) varies with the atomic number (Z). Nanoparticles with high atomic number have a dose-enhancing effect (8, 11, 12). By investigation of the biological mechanisms of high atomic number nanomaterials, theories such as cell cycle effects, DNA repair inhibition and mitochondrial dysfunction have been proposed to explain the biological process of radiation sensitisation of nanomaterials. The cell cycle is an important factor affecting radiosensitivity, with most cells exhibiting radioresistance in late S-phase and radiosensitivity in late G2 and mitotic phases; therefore, metal nanoparticles can improve the radiosensitivity of tumor cells by altering the cell cycle. IR treatment leads to mitochondrial dysfunction and induces the action of related molecules that increase the production of intracellular reactive oxygen species (ROS). Metal nanomaterials promote IR-induced oxidative stress, another important mechanism for their radiosensitization. Chow et al. (13) used Monte Carlo simulation algorithms to obtain how to maximize DER for nanoparticle-enhanced radiotherapy and demonstrated that gold nanoparticles are the most effective material in nanoparticle-enhanced radiation therapy. In addition, lower photon beam energy (6 MV), FFF photon beam, and higher nanoparticle concentration improve the DER of radiation therapy. Understanding the mechanisms of action can help to improve nanoparticle-enhanced radiotherapy and achieve better treatment outcomes.

The atomic number of metal nanoparticles is high, and has been shown to have a dose-enhancing effect on radiotherapy. Among them, gold nanoparticles have been the most extensively studied. AuNPs, with diameters of 1 to 100 nm, are widely used in materials, bioanalytical chemistry, industrial catalysis and medicine due to their high electron density, dielectric properties and catalytic properties, and their ability to bind to various biomolecules without affecting their biological activity (14). In the field of radiotherapy sensitization, there have been many reports in the literature both at home and abroad confirming the role of radiotherapy sensitization. As early as 2000, Herold et al. (15) conducted a sensitization study on AuNPs. Since then, Hainfeld et al. have investigated the sensitization of AuNPs in mammary tumor mice (16), mice with squamous cell carcinoma of the head and neck (17) and mice with malignant glioma in the brain (18) during radiotherapy. Bobyk et al. (19) actually evaluated the therapeutic efficiency of synchrotron stereotactic radiotherapy combined with loco-regional administration of gold nanoparticles for the treatment of orthotopic F98 gliomas in rats. The median survival time reached 41 days in the rats treated with the combination therapy, while the median overall survival time was 35 days in the rats irradiated alone, which equated to a significant 58% increase in life span. In addition, Zhang et al. (20) used a Monte Carlo simulation algorithm to find that the addition of 10^13^ gold nanospheres per cubic centimeter increased the absorbed radiation dose by 60%, mathematically demonstrating that AuNPs could enhance radiosensitivity. The exact mechanism of radiosensitization by gold nanoparticles is still under debate. The mainstream view is that their radiosensitizing effect is due to the increased photoelectron absorption of high atomic number materials when irradiated (21). Wang et al. (22) suggested that gold nanoparticles could achieve radiosensitization by modulating the cell cycle. Cells are the most radiosensitized in the G2/M phase (15). Zheng et al. (23) conducted a series of experiments to investigate the mechanism of AuNPs sensitization, and found that AuNPs could bind to DNA strands with and without AuNPs by electrostatic forces and adsorb a monolayer of DNA. This demonstrated that AuNPs could induce DNA breakage in tumor cells. Charnay Cunningham et al. (24) confirmed the radiosensitizing potential of AuNPs with proton radiotherapy by evaluating the radiosensitizing effect of AuNPs in combination with a 200 MeV proton beam.

Similar to gold nanomaterials, other metals such as silver, platinum, gadolinium and titanium have similar sensitizing effects on radiotherapy. Silver nanoparticles (AgNPs) combined with radiotherapy can prolong the survival time of glioma mice, which proves that they can enhance the radiosensitivity of human glioma cells *in vitro (* 25, 26). Liu et al. (27) found that in rats with malignant gliomas treated with silver nanoparticles, the mean survival time was significantly higher than the rest of the controls. Synergistic anti-proliferative and pro-apoptotic effects were also obtained. Porcel et al. (28) concluded that platinum nanoparticles (PtNPs) caused nearly 2-fold lethal DNA damage after radiation exposure. Hossain et al. (29) found that the radiosensitization effect of bismuth nanoparticles was stronger than that of gold and platinum nanoparticles under the same physicochemical conditions. Gadolinium (Z=64)-based nanoparticles are another commonly used radiosensitizers. One study found that in SQ20B tumor-bearing mouse model, combining the Gd-based nanoparticles with 10 Gy irradiation significantly delayed tumor growth (30). Zhang Li et al. (31) showed that tail vein injection of hyaluronic acid-functionalized gadolinium oxide nanoparticles (HA-Gd2O3) in combination with radiotherapy significantly inhibited the growth of mouse hepatocellular carcinoma cells compared with radiotherapy alone. The proliferation and cloning efficiency of HepG-2 cells were significantly inhibited by radiotherapy after intravenous tail injection of HA-Gd2O3 compared with radiotherapy alone. Titanium nanoparticles also have a sensitizing effect on radiotherapy, and Townley et al. (32) used human rhabdomyosarcoma cell lines RH30 and RD, and thymic carcinoma cell line MCF7 as targets. After radiotherapy, radiation energy was transferred to the TiO2 crystal structure, resulting in a high production of ROS that destroy cancer cells. Jiang et al. (33) found that palladium nanoparticles alone did not decrease the cell viability, indicating the excellent cytocompatibility of the nanoagents. Treatment of cancer cells with both X-rays and Pd NSs resulted in lower survival rates than cells treated with X-ray irradiation alone, indicating the radiosensitizing effect of Pd NSs. Alloying is an effective chemical method to adjust the properties of metal clusters. Recently, it has been demonstrated that Pt2 Au4 clusters exhibit peroxidase-like activity, which regulates tumor hypoxia and enhances the efficacy of radiotherapy through the sustained production of O2 by endogenous H2O2 decomposition (34). In addition to the above nanomaterials, metallic magnetic nanomaterial Zn/Fe2O4can also enhance radiotherapy sensitivity (35).

### 2.2 Inorganic non-metallic nanomaterials

The inorganic non-metallic nanomaterials also have the function of sensitizing radiotherapy. Carbon nanomaterials, which are isomers of carbon, are promising in the study of tumor radiosensitization due to their unique properties. Kleinauskas et al. (36) treated human prostate cancer cell lines LNCaP, Du145 and immortalized fibroblasts F11-hTERT with Carbon-core silver-shell nanodots, and the survival rate of normal cells F11-hTERT was significantly above than that of cancer cells LNCaP and Du145 after the same dose of radiation. The intrinsic mechanism may be due to the production of large amounts of ROS, which rupture lysosomes and release histone proteases, causing apoptosis. Ni et al. (37) found that C60 fullerenes induced cell membrane disruption and synergistic DNA damage after γ-ray irradiation in a study of mouse melanoma cell line B16 and human liver cancer cell line SMMU-7721.

Selenium (Se) nanoparticles have also been found to have a sensitizing effect on radiotherapy. Selenium nanoparticles both function as chemotherapeutic agents (38) and enhance the anti-tumor effects of X-rays by activating signaling pathways associated with ROS production, DNA cleavage, caspase-3 activation, mitochondrial damage and other apoptosis-inducing pathways. Yu et al. (39) co-cultured PEG decorated selenium nanoparticles (PEG-SENPs) with human cervical cancer Hela cells and mouse embryonic fibroblasts NIH3T3. After X-ray irradiation, 20 μM PEG-SENPs reduced the survival rate of Hela cells to 39%, while NIH3T3 cells were still 85% active even after the addition of 80 μM PEG-SENPs. In a retrospective study by Li et al. (40), it was found that among 18 patients with stage III/IV non-small cell lung cancer with local recurrence or metastasis who received multiple iodine particle implantation combined with external radiotherapy, the 1-year and 2-year survival rates were 62.5% and 32.7%, respectively, with a median survival of 31 months, compared with 6-9 months for radiotherapy and chemotherapy alone.

Nanodiamonds can also act as active nanoparticles that weaken the resistance of tumor cells to radiotherapy by promoting ROS production, damaging DNA and regulating the cell cycle (41). Hydrogenated nanodiamonds (H-NDs) have negative electron affinity that makes it highly reactive and positively charged with oxygen species and a positive charge in aqueous solutions. It can emit electrons after photon irradiation and may therefore enhance the effects of radiation on cancer cells. These studies suggest that the deleterious effects of DNA DSBs produced by NCS or ionizing irradiation can be amplified by H-NDs. H-NDs is not only expected to improve the treatment of radioresistant tumors, but may also reduce side effects by lowering the dose of radiotherapy for radiosensitive tumors. In summary, H-NDs is undoubtedly a valuable candidate radiosensitizer possibly associated with antisense molecular therapy.

### 2.3 Organic polymeric nanomaterials

Chitosan nanomaterials have a bidirectional regulatory effect on tumor cells and normal cells. Pan et al. (42) used heavy ion radiation alone as a control and found that at a nano-chitosan concentration of 500-1,000 mg/L, it significantly increased the radiosensitivity of human nasopharyngeal carcinoma KB cells, while increasing the tolerance of murine osteoblasts MC3T3⁃E1 to radiation damage. Chitosan can increase oxygen supply and improve intracellular oxygen levels, especially in hypoxic cells, while normal cells are well oxygenated. Chitosan can antagonize the damage caused by ROS-induced lipid peroxidation and enhance the radiation tolerance of normal tissues.

Dai et al. (43) developed a ruthenium-based metal-organic nanostructured radiotherapy sensitizer (ZrRuMn-MONs@mem) for the combined treatment of ROS and CO by increasing the direct absorption of radiation dose and facilitating the deposition of photons and electrons. In the present study, ruthenium metal-organic nanostructures show unique advantages in radiotherapy sensitization. Firstly, the presence of high Z elements enhances the absorption of X-rays and improves the production of ROS. Secondly, the special metal-organic nanostructures are able to enhance the efficiency of radiokinetic therapy by enabling energy and electron transfer through organic ligands. In addition, the domain-limited spatial structure plays an important role in confinement and conduction, increasing the chance of electron collisions with ground state electrons in the excited state and reducing electron losses.

The abundant blood flow, wide gap and poor structural integrity of blood vessel wall in solid tumor tissue, and lack of lymphatic reflux lead to the phenomenon of selective high permeability and retention of macromolecules and nanoparticles in some tissues, which is called ERP effect. Due to the EPR effect, nanomaterials of the right size can accumulate in tumor tissues and improve the sensitivity of tumors to radiotherapy through passive targeting (44, 45).

### 2.4 Chemotherapeutic drug nanoparticles

Currently, many small molecule compounds or chemotherapeutic agents also have a sensitizing effect on radiotherapy (46) such as catechin (47),adriamyci, paclitaxel (48–50), docetaxel (51–54), cyclopamine (55), Cisplatin and other platinum-based drugs (56–59), mitomycin C (60), selenocysteine (61), topotecan (62), camptothecin (63), histone deacetylase inhibitors (64), curcumin (65–67), tirapazamine (46, 68, 69), etanidazole (46), Arsenic trioxide (70), derivatives of selenium (71), NO (72–74). These chemotherapeutic agents with radiosensitizing effects are coupled with liposomes, proteins, polymers, dendrimers, exosomes, etc. to produce nanomedicines with radiosensitizing effects. In a study by Werner et al. (48) paclitaxel nanopolymeric micelles (Genexol-PM) were produced by polymerization of paclitaxel and measured (23.91±0.41) nm. After treatment with Genexol-PM, the sensitivity enhancement ratios (SER) of non-small cell lung cancer cell lines A549 and H460 were 1.12, 1.23, 1.03 and 1.12 at the small molecule and nano levels of paclitaxel, respectively, after X-ray irradiation. This indicates that paclitaxel nanoparticles have a better sensitization effect. At the same time, these nano-polymeric micelles can slowly release paclitaxel and enhance the synergistic effect of paclitaxel and radiotherapy. Cui et al. (75) made docetaxel⁃loaded nanoparticles (DOC⁃NPs) with an average particle size of 85 nm and used them to study gastric cancer cell lines BGC823, SGC7901, MKN45 and gastric mucosa cell line GES⁃1. After radiation treatment, the SER increased by 24%, 18% and 9% in the three gastric cancer cells, respectively, but only by 2% in GES⁃1 cells.

In addition, the relatively high concentrations of NO can also be used as an effective oxygen-depleted radiosensitizer. Fan et al. (72) modified NO donor (S-nitrosothiol) on upconverted nanoparticles to inhibit the growth of deep oxygen-depleted solid tumors by controlled release of NO under X-ray excitation.

### 2.5 Nano drug delivery system for loading radiosensitizers

Nano drug delivery systems are drug delivery systems that use nano technology as a carrier to disperse, encapsulate and adsorb drugs onto them, and are made with a particle size of 1 to 100 nm, which can increase drug absorption, improve drug targeting and slow release, increase the permeability of biological membranes, and reduce the toxic side effects of drugs. Nanodrug delivery systems can be loaded with Chinese and natural drugs, chemically synthesized drugs, peptide proteins and nucleotides (76, 77). More importantly, radioactive particles such as 223Ac(releasing A-particles), 131I, and 125I can be delivered precisely to tumor sites (9). The mechanism of controlled drug release using different types of nanocarriers is shown in **Figure 1C**. With the development of nanotechnology, nano based delivery systems show great potential for the delivery of radiosensitizers. Tian et al. (78) loaded the DNA double-strand repair inhibitor KU55933 into a high molecular polymer to produce nano-KU55933 and treated human lung cancer cell lines H460, A549, H23 and non-obese diabetic severe combined immunodeficient mice (NOD SCID white mice). After X-ray irradiation, this nanoparticle-loaded KU55933 was found to inhibit the DNA repair signaling pathway for a longer period of time than regular KU55933, while exhibiting lower skin toxicity. The presence of a large number of hypoxic cells in tumor cells is an important factor in the efficacy of tumor radiation therapy, and the more cells in the hypoxic region, the greater the likelihood of local postoperative recurrence. In a study of breast cancer cell lines MDA-MB-231 and of ZR-75-1, the number of oxygen-depleted cells in the tumor cells is a major factor affecting the effectiveness of radiation therapy. Jia et al. (79) used carbon nanotubes (CNTs) as carriers to load oxygen and modified them with folic acid to increase the dispersion and targeting of the drug delivery system to treat breast cancer cells in a hypoxic environment. Compared to the irradiated group alone, apoptosis-related proteins Bcl-2 and Survivin, hypoxia-inducible factor HIF-1α and radiation-related proteins Rad51 and Ku80 were all down-regulated, suggesting that folic acid-modified rare earth-modified oxygen-loaded carbon nanotubes could enhance the radiosensitivity of breast cancer cells.

**Figure 2:**
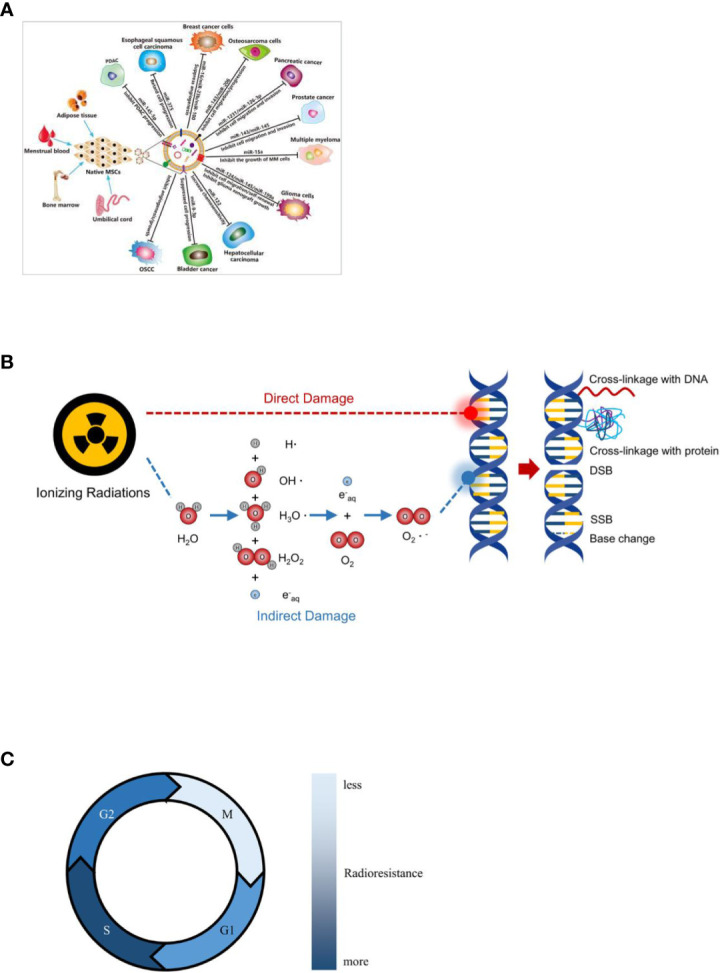
Mechanisms associated with nanoradiotherapy sensitization. **(A)** MSCs derived EVs for cancer therapy. This research was originally published in (83). Copyright(2022) Drug Delivery. **(B)** Direct and indirect effects of radiation with DNA. **(C)** Effect of cell cycle on radiosensitivity.

Specific active targeting is the most striking feature of nanodrug delivery systems. Nanotechnology has been used to construct nanodrug delivery systems that are coupled to antigens or antibodies and target genes specifically expressed by tumor cells, with specific targeting to that tumor cell. Khoshgard et al. (80) coupled folic acid with gold nanoparticles to form a nano-drug delivery system that was targeted to Hela cells and significantly improved the sensitivity of radiotherapy. Deng Wen et al. (81) prepared polyethyleneimine (PEI)-Fe3O4 magnetic nanoparticles (PEI-Fe3O4) encapsulated with miRNA-Survivin plasmids and used this novel nanotransfection vector to transfect the Survivin gene in CNE-2 cells of nasopharyngeal carcinoma to target and regulate the sensitivity of CNE-2 cells to radiation. This novel nano-transfection vector was found to be low in toxicity, non-immunogenicity, and it can significantly increase the apoptosis rate of CNE-2 cells compared with radiotherapy alone. Menon et al. (82) coupled the prostate cancer cell-permeable condensate R11 and the radiosensitizer NU7441 with poly (lactic-co-glycolic acid) (PLGA) into nanoparticles ((274 ± 80) nm) with sustained and slow release of radiosensitizer, which demonstrated good targeting and radiosensitization of prostate cancer PC3 cells. EVs loaded with a variety of therapeutic components such as tumor suppressor drugs, siRNAs, proteins, peptides, and conjugates exhibit significantly enhanced anti-tumor effects, so EVs can be used as an advanced and promising nanocavitor (83) (**Figure 2A**). In addition, the design and preparation of tumor-targeted modified EVs have greatly enhanced the specificity and effectiveness of tumor therapy, providing new ideas for tumor precision medicine.

## 3 Mechanisms and pathways of radiotherapy sensitization

With the development and advancement of molecular biology of tumors, researchers have tried to explain how the above-mentioned nano-sensitizers enhance the sensitivity to radiotherapy and investigate the mechanism of sensitization at the tissue level, cellular level and even at the molecular biology and genetic level. Current studies have mainly explored the physical and biochemical aspects of the mechanism of action of radiotherapy sensitization.

### 3.1 Physical perspective

In the physical sensitization, the strong X-ray absorption capacity of the material is mainly used to improve the ray absorption cross section, deposit high-energy ray energy, promote the generation of free radicals, directly enhance DNA damage, so as to improve the effect of radiotherapy.

DNA is the main target molecule in radiation therapy, and ionizing radiation usually damages DNA in two ways (**Figure 2B**). Firstly, it can directly ionize DNA molecules, resulting in various types of damage such as single or double strand breaks and cross-linking of bases and sugars. The second is the indirect reaction of high-energy radiation with water in the tissue to produce free radicals that bind to DNA, causing oxidation of the target molecule by electron transfer and inducing cellular DNA damage, leading to cellular damage or apoptosis. Many scholars believe that the localization of high-Z substances in the nucleus can produce more efficient DNA damage and radiobiotic effects, and cytoplasmic events are also important mechanisms leading to cell death (84, 85). Chun et al. (86) studied gold nanoparticles (GNPs) as radiation sensitizers leading to DNA damage during radiotherapy and concluded that the dose enhancement ratio (DER) was significantly correlated with GNP size, distance to DNA, and the photon energy. Under normal conditions, cells automatically repair the damaged DNA molecules to restore normal physiological function. In the presence of the reducing agent Glutathione (GSH), free radicals can be scavenged by hydrogen atom transfer, which acts as a radioprotector and prevents tumor cells from being killed. In the presence of an electrophilic sensitizer, on the one hand, it can take electrons from the target molecule DNA to oxidise it and prevent it from reabsorbing electrons for repair, resulting in potentially lethal chemical damage to the cell. On the other hand, it can also inhibit sulfhydryl compounds such as GSH in the cell, depleting the protective sulfhydryl groups in the cell and sensitizing it, thus increasing the sensitivity of the cell to radiation and improving the effectiveness of radiotherapy.

### 3.2 Biochemical perspective

Radiotherapy sensitizers can enhance the sensitivity of tumor cells to radiation by promoting reactive oxygen species (ROS) generation, regulating cell pathways and cell cycle, improving endogenous hypoxia in cells, inhibiting DNA repair, inhibiting tumor angiogenesis, and inhibiting autophagy. ROS mainly includes superoxide anion(O2•-), hydrogen peroxide (H2O2), singlet oxygen (1O2), hydroxyl radical (•OH) and so on. The DNA or proteins in cancer cells were damaged by the generated free radicals (87). Among them, H2O2 is a significant signaling molecule in tumor cells, which can promote the growth and development of tumor, and the increased H2O2 level is conducive to the stability of HIF. Since H2O2 is the most stable ROS in cells, the high concentration of cellular H2O2 can accelerates the production of various highly reactive oxygen species (HROS), such as •OH, hypochlorous acid (HOCl) and peroxynitrite (ONOO-). Next these HROS can promote the oxidative damage of proteins, membrane lipids and DNA. The tumor microenvironment (e. g. intratumoral oxygen content) has an important influence on the outcome of radiotherapy for tumors. 10%-50% oxygen-depleted cells are present in tumor tissue. Studies have shown that the radiosensitivity of cells irradiated in the presence of oxygen is about three times higher than in the absence of oxygen (88, 89). This means that the radiation dose required to kill hypoxic cells is much higher than that required to kill fully oxygenated cells. These hypoxic tumor cells are less likely to be killed because they are insensitive and resistant to radiation, resulting in metastasis or recurrence of tumor cells and ultimately failure of radiotherapy. Other factors may also affect the effectiveness of radiotherapy, for example, free radicals such as ROS generated during ionization are easily scavenged by reduced glutathione, reducing the killing effect of radiotherapy. Reduced effectiveness due to repair of potentially lethal damage to damaged cells is also a factor that affects cellular radiosensitivity. The radiation sensitivity of cells in different phases of the division cycle varies considerably (**Figure 2C**), with cells in the M and late G2 phases being the most sensitive and cells in the G1/S phase being three times more radioresistant than those in the G2/M phase (89); autophagy has a cytoprotective effect and reduces cytotoxicity and radiotherapy damage. Therefore, the biochemical perspective is to target the characteristics of the tumor microenvironment and to achieve sensitization radiotherapy through bioreduction, inhibition of damage repair, depletion of endogenous sulfhydryl groups, modulation of the cell cycle, radiosensitization of cell membranes, alteration of cellular oxygenation, inhibition of energy metabolism, inhibition of autophagy, induction of apoptosis, and radiation-induced gene therapy.

## 4 Clinical translation of nano-radiosensitizers

Nanomaterials and their associated nanomedicine with their ultra-small size and customisable multiple physicochemical properties are an emerging field for clinical biomedical that can be used in both diagnostic and therapeutic aspects of cancer. Over the past decades, the application of nanomaterials in the biomedical field has been widely and deeply developed, and some nanomaterials have already entered the clinical translation stage, such as liposomes, polymers, dendrimers, exosomes, gold nanoparticles, SiO2 nanoparticles and iron oxide nanoparticles. Here, we would like to analyze the current status of promising nano-radiosensitizers and their conversion. For example, nano-radiotherapy sensitizers that have entered preclinical or clinical trials include NBTXR3 and AGuIX.

NBTXR3, developed by the French company Nanobiotix. NBTXR3, of which the effective radiosensitizing component is HfO2 nanoparticles (32, 90–93) has entered clinical studies. HfO2 is relatively chemical inert in biological media, which reduces its biotoxicity and favors its biomedical applications. More importantly, Hf is a high Z atom (Z=72), so it can be used as a radiosensitizer (90), which can be achieved by physical mechanisms (93, 94). When HfO2 enters the tumor tissues and becomes activated, it can emit high energy electrons and increase the density of electrons deposited in the irradiated tissue, resulting in an increase in the radiation dose received by the tumor, while the dose passing through healthy tissues remains unchanged. This physical interaction between high-energy photons, HfO2 nanoparticles, and cancer cells can promote the production of cytotoxic free radicals, etc., which can damage DNA double-strands (90), thereby inhibiting tumor growth. In addition, Monte Carlo simulations also revealed a 9-fold increase in radiation dose to tissues receiving NBTXR3 compared with water exposure alone (32). The first human trial demonstrated that preoperative external irradiation of NBTXR3 produced encouraging radiological and pathological responses in patients with locally advanced soft tissue sarcomas of the extremities and trunk walls and was a technically feasible treatment (94). NBTXR3 is already in clinical studies, mainly for soft tissue sarcoma, head and neck tumors, prostate cancer, rectal cancer, liver cancer, oral cavity and throat cancer, with potential indications including esophageal cancer, malignant glioma and cervical cancer. Survival data from the American Society for Radiation Oncology (ASTRO) 2021 Annual Meeting for Nbtxr3 in head and neck cancer showed: median overall survival of 18.1 months and median progression-free survival of 10.6 months in evaluable patients (n=41); objective remission rates of 85.4% and complete remission rates of 63.4% were observed for target lesions. NBTXR3 is being tested in clinical studies to validate its efficacy in a variety of cancers, in addition to initiating clinical trials of checkpoint inhibitors in combination with immunization.

In addition, Tillement et al. discovered AGuIX, a nano-radiosensitizer, which can enhance the radiosensitivity of brain tumor cells (95, 96). The hydrodynamic diameter of this ultramicro-AguIX nanoparticle is less than 5 nm, indicating that it can be excreted through the kidney, avoiding biosafety concerns. AGuIX is composed of Gd chelated polysiloxane, and the interaction between Gd and X-rays enhances the efficacy of radiotherapy (96–99). In addition, AGuIX can accumulate in tumor tissues due to the EPR effect (100), further contributing to the efficacy of radiotherapy. To demonstrate the radiosensitizing effect of AGuIX in brain metastatic tumor cells, several phase I/II clinical trials on patients with brain metastatic tumors are ongoing. Also, the indications for AGuIX also include cervical cancer, liver cancer, lung cancer, esophageal cancer, and head and neck tumors (96, 101). This suggests that NBTXR3 and AGuIX have great potential for development and application. Ongoing clinical trials of radiosensitization with NBTXR3 and AGuIX are summarized in **Table 1**.

**Table 1:** Clinical translation of some nano-radiosensitizers.

Name	Conditions	Phase	Identifier
NBTXR3 Aguix	Head and neck squamous cell carcinoma Esophageal adenocarcinoma Lung non-small cell carcinoma Pancreatic ductal adenocarcinoma Metastatic malignant solid neoplasm Advanced cancers Head and neck squamous cell carcinoma Head and neck squamous Adult soft tissue sarcoma Adult soft tissue sarcoma Brain metastases Gynecological cancers Glioblastoma Brain metastases Brain metastases Lung tumors and pancreatic cancer Recurrent cancer	Phase III Phase I Phase I Phase I Phase I/II Phase I Phase II Phase I Phase II/III Phase I Phase II Phase I Phase I/II Phase II Phase I Phase I/II Phase II	NCT04892173 NCT04615013 NCT04505267 NCT04484909 NCT05039632 NCT03589339 NCT04862455 NCT01946867 NCT02379845 NCT01433068 NCT04899908 NCT03308604 NCT04881032 NCT03818386 NCT02820454 NCT04789486 NCT04784221

## 5 The challenge of nanomaterials as radiosensitizers

Although there have been some successes in the use of multifunctional nanomaterials for tumor sensitization, there are still many problems. There are a number of factors affecting the sensitization effect of radiotherapy, mainly including the following (**Figure 1B**): (1) Radiation energy intensity and dose. Differences in the killing effect of radiation on cells depending on energy intensity and dose. Ngwa et al. (102) suggested the sensitizing effect of nanogold on X-rays is better than that of γ-rays. The dose of radiation has a significant impact on the effectiveness of radiotherapy. Increasing the dose of radiation therapy can effectively control local tumors, but can cause serious side effects on healthy tissues. This is why it is essential to choose the right type of radiation and the right dose to achieve the best results. (2) Nanosensitizer composition and structure. The composition and structure determine its function and different sensitizers have different sensitizing effects. Hossain et al. (29) found that compared with gold and platinum nanoparticles, bismuth nanoparticles had stronger sensitization effect when the particle size, concentration and action site were the same. (3) Size and morphology of sensitizer. On the one hand, size is an important factor in determining the circulation time of nanomaterials in the blood. As a result, the toxicity of nanomaterials is related to their size and the sensitizing effect may vary depending on their size. For example, gold nanoparticles at 50 nm under 220 kVp excitation had a higher radiotherapy sensitization ratio (1.43) compared to gold nanoparticles at 14 and 74 nm (1.20 and 1.26) (103). On the other hand, the sensitization effect of the same radiotherapy sensitizer may vary from one form to another. Ma et al. (104) prepared three types of gold nanoparticles (GNPs), rods (GNRs) and stars (GNSs) of 50 nm in size, and modified them with PEG molecules. The order of cellular uptake of the three nanogold shapes was found to be GNP > GNSs > GNRs, with corresponding sensitization ratios of 1.62, 1.37 and 1.21, respectively, indicating that the shape of Au-based nanomaterials could affect the radiotherapeutic effect of tumor cells. (4) Sensitizer dose. The effect of concentration on radiotherapy dose enhancement is more significant than the effect of size (105). Increasing the concentration of gold nanoparticles leads to a reduction in the number of cells, as higher concentrations mean that more gold atoms interact with X-rays and more X-ray energy can be deposited. However, higher concentrations of nanomaterials can increase the risk of cytotoxicity. There is therefore a trade-off between the radiation dose enhancement effect and the permitted concentration of toxicity. (5) Sensitizer surface modification and functionalization. Different functionalized groups (e.g. PEGs, carboxyl groups, amino groups, thiols, drugs, DNA, lipids, sugars, antibodies, peptides, organic small molecules) are modified on the surface of the nanoparticles to give them a variety of properties (106). Surface modification may improve biocompatibility, cellular uptake, targeting ability, accumulation, surface charge, biological half-exclusion period, toxicity, etc., leading to better sensitization for radiotherapy. For example, the surface modification of gold nanoparticles with GSH261 or PEG46 helps to evade uptake by the reticuloendothelial system. (6) Tumor cell type. Numerous studies have shown that radiosensitization is cell selective and that the cytotoxicity of the same nanomaterial varies between cell types. For example, glucose-modified gold nanoparticles do not enhance the radiosensitivity of human diploid fibroblasts, but can increase the radiosensitivity of human prostate cancer cells (107). This may be related to the different levels of uptake of the same nanomaterial by different cells, or it may be related to the cell cycle in which the cells are dividing. Some cancer cells are highly proliferative and have rapid DNA replication, resulting in S and G0 stage cells that are radioresistant, while less actively dividing cells, most of which are in the M and G2 stage of the cell cycle, are most sensitive to radiation.

**Figure 3:**
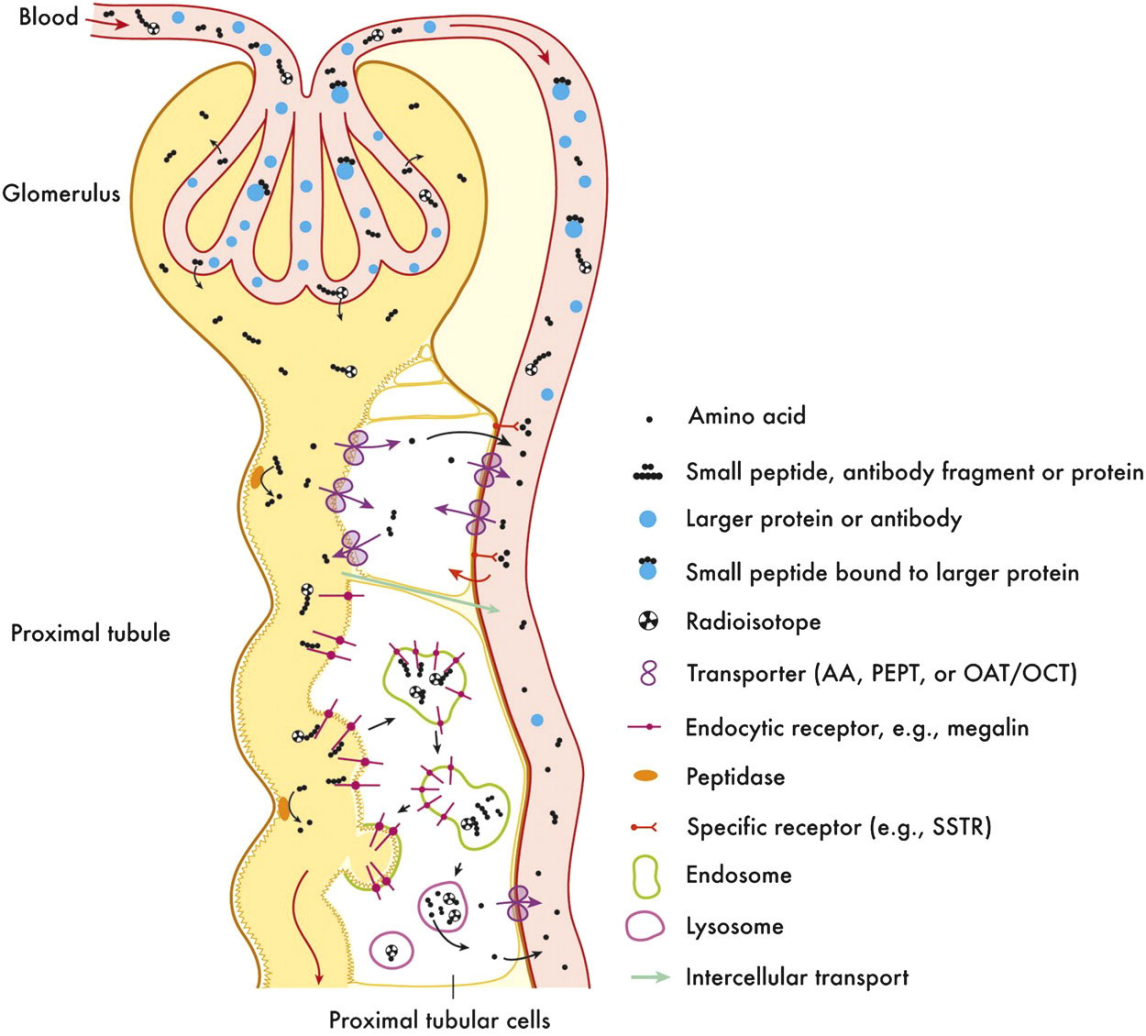
Clearance and re-absorption of peptides and small molecules in kidney. This research was originally published in (108). Copyright (2010) Journal of nuclear medicine.

The special characteristics of medical nanomaterials dictate that their bioeffectiveness and safety should be given top priority, and how to improve the biosafety of nano-radiotherapy sensitizers is a key issue that needs to be addressed. We must consider its acute and long-term toxicity. To date, few acute toxicities have been observed in *in vivo* studies of radiosensitizers. However, long-term effects are more difficult to assess. Biodistribution studies typically indicate a burden on liver, kidneys and spleen and long-term effects on these organs may be possible (**Figure 3**). The ideal nano-radiotherapy sensitizer should have biodegradable components, a renal metabolizable size, a suitable half-life, low toxic effects on healthy tissue, and a good sensitizing effect on radiotherapy, particularly in terms of both sensitizing tumor tissue and reducing toxic effects on normal tissue. In addition, a further difficulty hindering the clinical development of nanoparticles is the difficulty in synthesizing identical nanoparticles quickly, accurately and reproducibly due to the fact that systematic parallel screening of the numerous properties of nanoparticles is still difficult. At the same time, the complexity of chemical manufacturing and control (CMC), good manufacturing practice (GMP) and other aspects of the process of moving from pre-clinical to clinical to commercialization has gradually increased. The process of translation from the laboratory to the clinic is often accompanied by optimization of parameters and even changes in methodology, so it is crucial to consider scaling issues when designing nanoparticles early on. In order to make nano-radiosensitizers suitable for clinical translation, rational pre-clinical protocols are needed and guidelines and standardization for the design and manufacture of nano-radiosensitizers are established. It is believed that in the near future, all these issues will be resolved, providing a scientific basis for the use of nanomaterials and nanotechnology in clinical radiotherapy for tumors.

## 6 Conclusions and future perspectives

From the earliest “free radical damage and fixation” strategies to gene regulation, from chemical substances to biomolecules to nanomaterials, radiosensitizers have been developed for decades. Although small molecules, macromolecules and nanomaterials have been developed, the research is still inadequate to meet the clinical needs of radiotherapy, and therefore more effective radiosensitizing agents can be further developed for clinical selection by addressing new targets and mechanisms of radiosensitization. The clinical application of existing nano-radiosensitizers has not been fully confirmed, and other techniques, such as molecular structure analysis, molecular cloning techniques and bioinformatics analysis, need to be flexibly applied to further optimizing radiosensitizers, discover nanomaterials with low cytotoxicity, good biocompatibility and easy functionalization and make them more effective clinical aids. But it is no doubtable that nanomaterials are a kind of very promising materials that can help to promote the development of radiotherapy. We believe that nanotechnologies will be applied in clinical practice in the near future and finally benefit mankind. This review examines the mechanisms of action and factors affecting the main types of nano-radiosensitizers, and provides an outlook on their future development and application.

## Author contributions

SY conceived and supervised the study. XS and ZS consulted the literature and wrote the manuscript. LL and LZ provided critical revision. All authors contributed to the article and approved the submitted version.
